# mRNA expression of diacylglycerol kinase isoforms in insulin-sensitive tissues: effects of obesity and insulin resistance

**DOI:** 10.14814/phy2.12372

**Published:** 2015-04-06

**Authors:** Louise Mannerås-Holm, Henriette Kirchner, Marie Björnholm, Alexander V Chibalin, Juleen R Zierath

**Affiliations:** 1 Department of Molecular Medicine and Surgery, Karolinska InstitutetStockholm, Sweden; 2 Department of Physiology and Pharmacology, Karolinska InstitutetStockholm, Sweden

**Keywords:** Adipose tissue, liver, skeletal muscle

## Abstract

Diacylglycerol kinase (DGK) isoforms regulate signal transduction and lipid metabolism. DGK*δ* deficiency leads to hyperglycemia, peripheral insulin resistance, and metabolic inflexibility. Thus, dysregulation of other DGK isoforms may play a role in metabolic dysfunction. We investigated DGK isoform mRNA expression in extensor digitorum longus (EDL) and soleus muscle, liver as well as subcutaneous and epididymal adipose tissue in C57BL/6J mice and obese and insulin-resistant *ob/ob* mice. All DGK isoforms, except for DGK*κ*, were detectable, although with varying mRNA expression. Liver DGK expression was generally lowest, with several isoforms undetectable. In soleus muscle, subcutaneous and epididymal adipose tissue, DGK*δ* was the most abundant isoform. In EDL muscle, DGK*α* and DGK*ζ* were the most abundant isoforms. In liver, DGK*ζ* was the most abundant isoform. Comparing obese insulin-resistant *ob/ob* mice to lean C57BL/6J mice, DGK*β*, DGK*ι*, and DGK*θ* were increased and DGK*ε* expression was decreased in EDL muscle, while DGK*β*, DGK*η* and DGK*θ* were decreased and DGK*δ* and DGK*ι* were increased in soleus muscle. In liver, DGK*δ* and DGK*ζ* expression was increased in *ob/ob* mice. DGK*η* was increased in subcutaneous fat, while DGK*ζ* was increased and DGK*β*, DGK*δ*, DGK*η* and DGK*ε* were decreased in epididymal fat from *ob/ob* mice. In both adipose tissue depots, DGK*α* and DGK*γ* were decreased and DGK*ι* was increased in *ob/ob* mice. In conclusion, DGK mRNA expression is altered in an isoform- and tissue-dependent manner in obese insulin-resistant *ob/ob* mice. DGK isoforms likely have divergent functional roles in distinct tissues, which may contribute to metabolic dysfunction.

## Introduction

Increased accumulation of lipid intermediates, such as triglycerides, diacylglycerol (DAG), ceramides, and long-chain fatty acid coenzyme A, contribute to the development of insulin resistance (Erion and Shulman 2010; Samuel and Shulman 2012; Zhang et al. 2013). Ectopic accumulation of specific lipid metabolites (diacylglycerols and/or ceramides) in liver and skeletal muscle may be caused by increased fatty acid delivery/synthesis when the adipose tissue storage capacity is exceeded and/or decreased mitochondrial fatty acid oxidation (Erion and Shulman 2010; Zhang et al. 2013). Increased intramuscular DAG activates a serine/threonine kinase signaling pathway, including protein kinase C (PKC), which results in serine phosphorylation of insulin receptor substrate (IRS) -1, inhibition of IRS-1 tyrosine phosphorylation, and reduced insulin-stimulated glucose uptake and metabolism (Samuel and Shulman 2012). In liver, increased DAG content reduces insulin-stimulated glycogen synthesis and decreases the suppression gluconeogenesis (Samuel and Shulman 2012). Thus, altered activity and/or abundance of enzymes involved in lipid metabolism influences the accumulation of different lipid intermediates and impinges upon intracellular signaling events.

Diacylglycerol kinases (DGKs) control the level of two important lipid messengers: DAG and phosphatidic acid (PA). DGKs catalyze a reaction that removes DAG, by converting this lipid to PA at the plasma membrane, endoplasmic reticulum and nucleus, and thereby terminates DAG-derived signals (Shulga et al. 2011). Consequently, DGK isoform expression may be altered in obesity and type 2 diabetes. Ten mammalian DGK isoforms have been classified into five subgroups based on different regulatory domains in their primary structure, although all isoforms have a catalytic domain and at least two C1 domains (Shulga et al. 2011; Sakai and Sakane 2012). DGK isoforms are unique, not only structurally, but also in their expression pattern, subcellular localization, regulatory mechanisms and DAG preferences, suggesting isoform-specific functional roles. While DGK isoform expression has been surveyed in various tissues including in mouse and rat reproductive organs (Toya et al. 2005; Shionoya et al. 2015), immune cells during an inflammatory reaction (Yamamoto et al. 2014), rat retina (Hozumi et al. 2013), lung (Katagiri et al. 2005), and regenerating liver (Nakano et al. 2012), and human failing hearts (Bilim et al. 2011), mRNA expression of DGK isoforms in tissues important for metabolic homeostasis in the context of type 2 diabetes and obesity is unknown.

DGK isoforms regulating wide variety of physiological processes including growth, metabolism, proliferation, immunological and neural development (Topham 2006; Krishna and Zhong 2013; Ishisaka and Hara 2014; Shirai and Saito 2014; Yamamoto et al. 2014). DGK*δ* expression is decreased in skeletal muscle from type 2 diabetic patients and haploinsufficiency (DGK*δ*^+/−^) in mice leads to insulin resistance and late-onset obesity (Chibalin et al. 2008). However, the role of other DGK isoforms in the development of metabolic disorders, including insulin resistance and obesity, is largely unknown. Here, we determined the tissue-specific mRNA expression of DGK isoforms in insulin-sensitive tissues including skeletal muscle, liver and two separate adipose tissue depots (subcutaneous and epididymal). We also explore the effect of severe obesity and insulin resistance on DGK isoform mRNA expression by comparing tissue-specific profiles between obese *ob/ob* (B6.V-Lep^Ob^/J) and lean C57BL/6J mice.

## Material and Methods

### Animals

Male lean C57BL/6J and obese *ob/ob* (B6.V-Lep^Ob^/J) mice were purchased from Charles River Laboratories (Italy). Animals were maintained in a temperature- and light-controlled environment (12-h light, 12-h dark cycle) and had free access to water and food. At 12 weeks of age, fed mice were anesthetized via intraperitoneal injection of 2.5% *Avertin* (0.02 mL/g body weight). Skeletal muscles (extensor digitorum longus (EDL) and soleus), adipose tissues (subcutaneous and epididymal) and liver were harvested from lean C57BL/6J (body weight 24.4 ± 0.2 g) and obese *ob/ob* (body weight 47.7 ± 1.2 g) and clamp-frozen in liquid nitrogen and stored for mRNA analysis. The regional animal ethics committee of Northern Stockholm approved all experimental procedures.

### RNA isolation, cDNA synthesis and qPCR

Tissues were homogenized using the TissueLyser (Qiagen, Hilden, Germany). Total RNA in adipose tissue and liver was isolated using RNeasy Lipid Tissue Mini Kit and RNeasy Mini Kit (Qiagen), respectively. A deoxyribonuclease I (QIAGEN) digestion step was included to eliminate DNA contamination. Total RNA in skeletal muscle was isolated using TRIzol reagent (Invitrogen Life Technologies Ltd, Paisley, UK), according to the manufacturer's protocol. RNA concentration and purity were determined with a Nanodrop ND-1000 spectrophotometer (Nano-Drop Technologies, Wilmington, DE, USA) and all samples had a 260/280 and a 260/230 ratio >2.0 indicating pure RNA. RNA integrity was verified with the Experion (Bio-Rad Laboratories, Hercules, CA) and all samples had a RQI value >8 indicating intact RNA. cDNA was synthesized from 1 or 2 *μ*g total RNA in a final reaction volume of 20 *μ*L using the High Capacity cDNA Reverse Transcription Kit (Applied Biosystems, Stockholm, Sweden), according to the manufacturer's protocol.

### Real-time qPCR

Real-time qPCR was performed in a StepOnePlus Realtime qPCR System (Applied Biosystems) using Taqman Gene Expression Assays (Table1). Multiplex qPCR was performed in duplicate using 20 ng cDNA mixed with TaqMan Gene Expression Master Mix (Applied Biosystems) in a total volume of 20 *μ*L. Cycling conditions were as follows: 95°C for 10 min, 40 cycles at 95°C for 15 sec and 60°C for 1 min.

**Table 1:** Taqman gene expression assays

Symbol	Name	Taqman Assay ID
Target genes		
*Dgka*	Diacylglycerol kinase, alpha (*α*)	Mm00444048_m1^*^
*Dgkb*	Diacylglycerol kinase, beta (*β*)	Mm00618478_m1^*^
*Dgkg*	Diacylglycerol kinase, gamma (*γ*)	Mm00446756_m1^*^
*Dgkd*	Diacylglycerol kinase, delta (*δ*)	Mm00617404_m1^*^
*Dgkh*	Diacylglycerol kinase, eta (*η*)	Mm01312241_m1^*^
*Dgkk*	Diacylglycerol kinase, kappa (*κ*)	Mm01340751_m1^*^
*Dgke*	Diacylglycerol kinase, epsilon (*ε*)	Mm00444676_m1^*^
*Dgkz*	Diacylglycerol kinase, zeta (*ζ*)	Mm00661896_m1^*^
*Dgki*	Diacylglycerol kinase, iota (*ι*)	Mm01159464_m1^*^
*Dgkq*	Diacylglycerol kinase, theta (*θ*)	Mm01198794_m1^*^
Reference genes		
*Rn18S*	18S rRNA	Mm03928990_g1^*^
*Actb*	Actin, beta	Mm00607939_s1^*^
*B2 m*	Beta-2-microglobulin	Mm00437762_m1^*^
*Gapdh*	Glyceraldehyde-3-phosphate dehydrogenase	Mm99999915_g1^*^
*Gusb*	Beta-glucuronidase	Mm00446953_m1
*Hprt1*	Hypoxanthine-guanine phosphoribsyltransferase	Mm00446968_m1^*^
*Pgk1*	Phosphoglycerate kinase 1	Mm00435617_m1^*^
*Ppia*	Peptidylprolyl isomerase A, cyclophilin A	Mm02342429_g1^*^
*Rplp0*	Ribosomal protein, large, P0	Mm01974474_gH^*^
*Tbp*	TATA-box-binding protein	Mm00446973_m1^*^

*The TaqMan gene expression assay with the best coverage for that specific gene.

Data were analyzed with Step One Software v2.1 (Applied Biosystems). Relative gene expression was calculated with the ΔΔC_q_ method (Livak and Schmittgen 2001) and expressed as fold changes compared with DGK*ε* mRNA in C57BL/6J mice. Ten candidate reference genes (Table1) were validated with NormFinder algorithm incorporated into the *GenEx* software (MultiD Analyses AB, Gothenburg, Sweden). The relative expression was normalized in EDL against the reference genes Gusb, Hprt1 and Ppia, in soleus against the reference genes 18S, Actb, Hprt1, Ppia, Rplp0 and Tbp, in liver against the reference genes Actb, B2 m, and Ppia, in subcutaneous adipose tissue against the reference genes B2 m, Pgk1, Ppia, and Tbp, and in epididymal adipose tissue against the reference genes Pgk1 and Ppia. Isoforms with a C_q_ > 35 were considered below the limit of detection.

### Statistics

Results are presented as mean ± SEM. Differences in gene expression between C57BL/6J and *ob/ob* mice were determined by Student's *t*-test on logarithmic transformed data. Significance was accepted at *P *<* *0.05.

## Results

### Tissue expression profile in C57BL/6J mice

**Figure 1:**
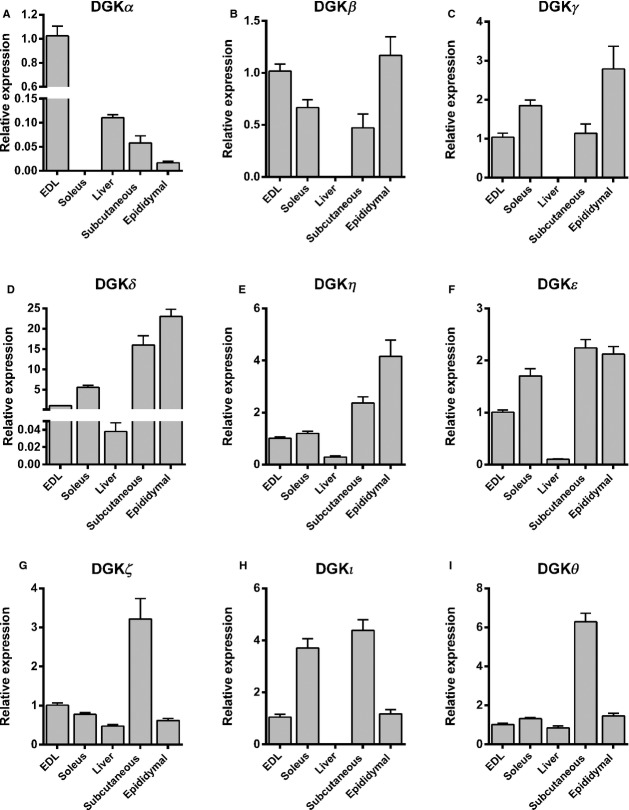
DGK isoform expression profile. mRNA expression of (A) DGK*α*, (B) DGK*β*, (C) DGK*γ*, (D) DGK*δ*, (E) DGK*η*, (F) DGK*ε*, (G) DGK*ζ*, (H) DGK*ι* and (I) DGK*θ* in EDL muscle, soleus muscle, liver, subcutaneous adipose tissue, and epididymal adipose tissue from C57BL/6J mice. Data are presented relative to respective mRNA in EDL muscle. Results are mean ± SEM. *n* = 8–10.

None of the reference genes were stably expressed across all investigated tissues (Table2). Therefore, data are presented as C_q_ values (Table3) and normalized against gene expression of each specific DGK isoform in EDL muscle (Fig.1) for the comparison between isoforms in different tissues of C57BL/6J mice.

**Table 2:** mRNA expression of reference genes in insulin-sensitive tissues from C57BL/6J mice

Gene	EDL	Soleus	Liver	Subcutaneous	Epididymal
Rn18S	NA	9.66 ± 0.06	NA	8.83 ± 0.12	NA
Actb	NA	23.84 ± 0.07	21.95 ± 0.10	18.18 ± 0.23	NA
B2m	24.65 ± 0.12	24.61 ± 0.08	20.25 ± 0.08	19.86 ± 0.14	NA
Gapdh	NA	19.79 ± 0.05	NA	20.56 ± 0.15	19.47 ± 0.14
Gusb	29.36 ± 0.12	29.17 ± 0.09	NA	26.49 ± 0.12	NA
Hprt1	27.58 ± 0.07	27.86 ± 0.07	NA	25.45 ± 0.12	24.93 ± 0.10
Pgk1	22.88 ± 0.09	25.14 ± 0.08	25.09 ± 0.07	25.51 ± 0.11	26.20 ± 0.11
Ppia	24.93 ± 0.08	25.61 ± 0.06	21.44 ± 0.09	21.93 ± 0.12	20.92 ± 0.11
Rplp0	22.35 ± 0.06	21.98 ± 0.10	NA	20.25 ± 0.20	NA
Tbp	NA	31.56 ± 0.10	NA	30.85 ± 0.15	NA

Data are presented as C_q_ values and expressed as mean ± SEM. NA, not applicable.

**Table 3:** DGK isoform mRNA expression in insulin-sensitive tissues from C57BL/6J mice

Gene	EDL	Soleus	Liver	Subcutaneous	Epididymal
DGK*α*	27.6 ± 0.1	ND	30.8 ± 0.1	32.3 ± 0.5	33.8 ± 0.3
DGK*β*	32.0 ± 0.1	32.7 ± 0.2	ND	33.8 ± 0.5	31.9 ± 0.2
DGK*γ*	33.2 ± 0.1	32.4 ± 0.1	ND	33.2 ± 0.2	31.9 ± 0.3
DGK*δ*	29.2 ± 0.1	26.8 ± 0.1	34.4 ± 0.4	25.4 ± 0.2	24.8 ± 0.1
DGK*η*	31.8 ± 0.1	31.6 ± 0.1	33.7 ± 0.2	30.6 ± 0.1	29.9 ± 0.3
DGK*κ*	ND	ND	ND	ND	ND
DGK*ε*	29.7 ± 0.1	28.9 ± 0.1	33.0 ± 0.1	28.5 ± 0.1	28.6 ± 0.1
DGK*ζ*	27.4 ± 0.1	27.8 ± 0.1	28.5 ± 0.1	25.9 ± 0.3	28.1 ± 0.1
DGK*ι*	34.7 ± 0.2	32.9 ± 0.1	ND	32.7 ± 0.2	34.0 ± 0.6
DGK*θ*	33.2 ± 0.1	32.8 ± 0.1	33.5 ± 0.2	30.6 ± 0.1	32.7 ± 0.1

Data are presented as C_q_ values and expressed as mean ± SEM. ND, not detected.

The DGK*α* gene was highly expressed in EDL muscle, but not expressed in soleus muscle and lowly expressed in liver and adipose tissue (Fig.1A). DGK*β* and DGK*γ* were not expressed in liver, while levels were similar between skeletal muscle and adipose tissues (Fig.1B and C). DGK*δ* and DGK*η* were expressed at higher levels in adipose tissues compared to skeletal muscle, with very low levels in liver (Fig.1D and E). A similar expression pattern was observed DGK*ε* (Fig.1F). The expression profile for DGK*ζ* and DGK*θ* between tissues was similar (Fig.1G and I); however a threefold and sixfold higher expression, respectively, was observed in subcutaneous adipose tissue, and similar levels were observed between skeletal muscle, liver and epididymal adipose tissue. Overall, mRNA expression of DGK*ζ* was higher than DGK*θ* (Table2). DGK*ι* was 4-fold higher in soleus and subcutaneous adipose tissue compared to EDL and epididymal adipose tissue, while not detected in liver (Fig.1H).

We next compared the relative isoform expression within each tissue. In EDL muscle, DGK*α* and DGK*ζ* had the highest expression level (Table3). While DGK*α* was not expressed in soleus muscle, DGK*δ* was the highest isoform expressed (Table3 and Fig.1). In general the expression level for all DGK isoforms was low in liver compared to other tissues, with DGK*β*, DGK*γ*, DGK*κ* and DGK*ι* were under the detection limit of the assay (Table3 and Fig.1). The expression level of the DGK isoforms also differed between the two adipose tissue depots analyzed; DGK*β*, DGK*γ*, DGK*δ* and DGK*η* were more highly expressed in epididymal adipose tissue as compared to subcutaneous adipose tissue with the inverse noted for DGK*α*, DGK*ζ*, DGK*ι* and DGK*θ* (Table3 and Fig.1). DGK*δ* had the highest expression in both adipose depots (Table3). DGK*ζ* was the only isoform with a C_q_ <  30 in all investigated tissues (Table3). DGK*β*, DGK*γ*, DGK*η*, DGK*ι* and DGK*θ* were expressed at low levels in all tissues (C_q_ > 30; Table3) and DGK*κ* was not detected in any of the investigated tissues.

### Tissue expression profile in C57BL/6J and *ob/ob* mice

**Figure 2:**
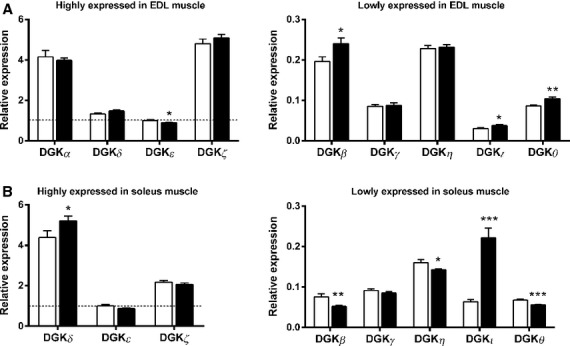
mRNA expression of DGK isoforms in skeletal muscle from C57BL/6J mice (white bars) and *ob/ob* mice (black bars). (A) Highly expressed (left panel) and lowly expressed (right panel) DGK isoforms in EDL muscle; (B) highly expressed (left panel) and lowly expressed (right panel) DGK isoforms in soleus muscle; Data are normalized to reference genes (see Material and Methods) and presented relative to DGK*ε* mRNA in tissues from C57BL/6J mice. Results are mean ± SEM. *n* = 8–10. *P < 0.05, **P < 0.01, ***P < 0.001.

**Figure 3:**
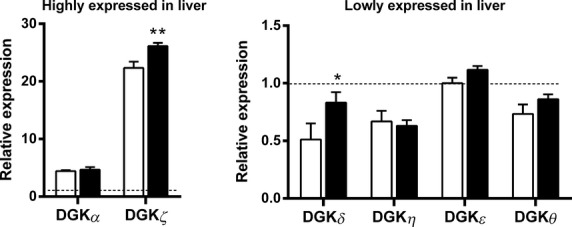
mRNA expression of DGK isoforms in liver from C57BL/6J mice (white bars) and *ob/ob* mice (black bars). Highly expressed (left panel) and lowly expressed (right panel) DGK isoforms in liver. Data are normalized to reference genes (see Material and Methods) and presented relative to DGK*ε* mRNA in tissues from C57BL/6J mice. Results are mean ± SEM. *n* = 8–10. *P < 0.05, **P < 0.01.

**Figure 4:**
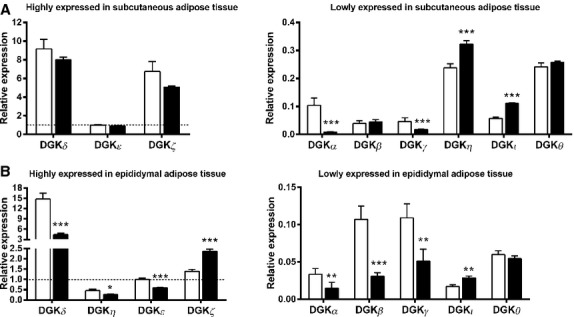
mRNA expression of DGK isoforms in subcutaneous and epididymal adipose tissue from C57BL/6J mice (white bars) and *ob/ob* mice (black bars). (A) Highly expressed (left panel) and lowly expressed (right panel) DGK isoforms in subcutaneous adipose tissue; (B) Highly expressed (left panel) and lowly expressed (right panel) DGK isoforms in epididymal adipose tissue. Data are normalized to reference genes (see Material and Methods) and presented relative DGK*ε* mRNA in tissues from C57BL/6J mice. Results are mean ± SEM. *n* = 8–10. *P < 0.05, **P < 0.01, ***P < 0.001.

Several DGK isoforms were differentially expressed between *ob/ob* and C57BL/6J mice. In EDL muscle, DGK*ζ* was the predominant isoform, followed by DGK*α*, DGK*δ* and DGK*ε* (Fig.2A). Moreover, expression of DGK*β*, DGK*ι* and DGK*θ* was increased, whereas DGK*ε* was decreased in EDL muscle from *ob/ob* compared to C57BL/6J mice (Fig.2A). In soleus muscle, DGK*δ* was the predominant isoform followed by DGK*ζ* and DGK*ε* (Fig.2B). Expression of DGK*δ* and DGK*ι* was increased, whereas DGK*β*, DGK*η*, and DGK*θ* were decreased in soleus of *ob/ob* compared to C57BL/6J mice (Fig.2B). In liver, DGK*ζ* was the predominant isoform followed by DGK*α* (Fig.3). Liver DGK*δ* and DGK*ζ* expression was increased in *ob/ob* compared to C57BL/6J mice (Fig.3). In subcutaneous adipose tissue, DGK*δ* was the predominant isoform followed by DGK*ζ* (Fig.4A). The DGK*η* and DGK*ι* expressions were increased and the DGK*α* and DGK*γ* expressions were decreased in subcutaneous adipose tissue of *ob/ob* compared to C57BL/6J mice (Fig.4A). In epididymal adipose tissue, DGK*δ* was the predominant isoform followed by DGK*ζ* (Fig.4B). DGK*ζ* and DGK*ι* expression was increased and DGK*α*, DGK*β*, DGK*γ*, DGK*δ*, DGK*η*, and DGK*ε* expression was decreased in epididymal adipose tissue of *ob/ob* compared to C57BL/6J mice (Fig.4B).

## Discussion

We first surveyed the DGK isoform expression profile in skeletal muscle, adipose tissue and liver from lean mice. Overall we found the DGK isoform expression profile was similar between glycolytic EDL and oxidative soleus muscle, except in the case of DGK*α* where mRNA expression was undetectable in soleus muscle. In liver, DGK*ζ* was the predominant isoform, with comparable levels of DGK*θ* and DGK*δ*, confirming an earlier report (Shulga et al. 2013). Of note, expression of DGK*β*, DGK*γ*, DGK*κ* and DGK*ι* in this tissue was minimal or not detected. In subcutaneous and epididymal adipose tissue, isoform-specific DGK expression patterns were also observed. While DGK*ζ* was the predominant isoform in liver, DGK*δ* was the predominant isoform in subcutaneous and epididymal adipose tissue, consistent with earlier reports (Lowe et al. 2013; Shulga et al. 2013). Likewise, DGK*δ* was highly expressed in skeletal muscle, consistent with human RNA-seq expression data (Krupp et al. 2012). Isoforms in the same subfamily did not necessarily share same expression profile and isoforms from different subfamilies were expressed at similar magnitude, further highlighting the tissue-specific roles of DGK isoforms.

Several genetically modified DGK mouse models have been generated (Rodriguez de Turco et al. 2001; Zhong et al. 2003; Regier et al. 2005; Crotty et al. 2006; Olenchock et al. 2006; Chibalin et al. 2008; Shirai et al. 2010), each demonstrating distinct phenotypic traits, further implying functional diversity in DGK isoform-specific signaling (Shirai and Saito 2014). Given that both the substrate (DAG) and the product (PA) of the reaction catalyzed by DGK serve as metabolic intermediates and potent signaling molecules, an imbalance in these substrates may have an impact on cell growth and metabolism. Ectopic intracellular accumulation of DAG is associated with insulin resistance in skeletal muscle and liver (Erion and Shulman 2010; Samuel and Shulman 2012; Zhang et al. 2013; Zachariah Tom et al. 2014). Consequently, an imbalance in expression of DGKs may have a role in the pathogenesis of various metabolic disorders including type 2 diabetes, obesity, and nonalcoholic fatty liver disease. Thus, we next determined the expression of DGK isoforms in obese insulin-resistant *ob/ob* mice.

DGK*α*, DGK*β* and DGK*γ* belong to the type I subgroup of DGKs, which have a calcium-binding EF hand motif (Shulga et al. 2011). DGK*α* is abundant in T cells and has a role in T-cell activation and T-cell anergy (Olenchock et al. 2006). In healthy humans, DGK*α* mRNA levels are comparable between adipose tissue, liver and skeletal muscle, DGK*γ* mRNA is increased in skeletal muscle compared to liver and adipose tissue, whereas DGK*β* is minimally expressed in these tissues (Krupp et al. 2012). DGK*α* and DGK*γ* are highly expressed in mouse pancreatic *β*-cells and diminished levels of these isoforms attenuates insulin secretion (Kurohane Kaneko et al. 2013), implying a role in metabolic disease. Indeed, mRNA of these isoforms was reduced in subcutaneous and epididymal adipose tissue of *ob/ob* mice, but the functional significance is unknown. Genetic studies have linked DGK*β* to diabetes risk. A meta-analysis of 21 genome-wide association studies found an association between the loci containing DGK*β* (DGKB-TMEM195) and type 2 diabetes (Dupuis et al. 2010). Even though this isoform was lowly expressed, DGK*β* mRNA was increased EDL muscle, and concomitantly decreased in soleus muscle and epididymal adipose tissue from *ob/ob* mice. Although DGK*β* may be involved in type 2 diabetes, knockout mice show attention-deficit behavior and a hyperactive phenotype, implicating a predominant role in neurological, rather than metabolic disorders (Ishisaka et al. 2012).

The members of type II DGKs, DGK*δ*, DGK*η*, and DGK*κ*, have pleckstrin homology domains and sterile *α* motifs (Sakai and Sakane 2012). We found that DGK*δ* and DGK*η* mRNA was readily detected in all insulin-sensitive tissues, whereas DGK*κ* was undetected. RNA-Seq data show DGK*κ* is lowly expressed in most tissues, with the highest expression noted in testes and brain (Krupp et al. 2012). In contrast, DGK*δ* is widely expressed in insulin-sensitive tissue (Chibalin et al. 2008; Krupp et al. 2012) and plays a major role in regulating the synthesis of a broad range of lipid species (Shulga et al. 2013). DGK*δ* also increases lipid synthesis by promoting de novo fatty acid synthesis (Shulga et al. 2013). DGK*δ*^+/−^ mice have increased DAG content in skeletal muscle, leading to impaired peripheral insulin sensitivity and age-dependent obesity (Chibalin et al. 2008). Here we report DGK*δ* is highly expression in skeletal muscle and adipose tissue, and to a lower extent in liver, consistent with a previous study (Lowe et al. 2013). Total DGK activity is reduced in skeletal muscle and adipose tissue, but not liver from DGK*δ*^+/−^ mice (Chibalin et al. 2008), indicating DGK*δ* plays a major role in peripheral tissues and a minor role in liver. In *ob/ob* mice, DGK*δ* mRNA was increased in liver and soleus muscle, decreased in epididymal adipose tissue and unaltered in subcutaneous adipose; further highlighting the impact of metabolic disease on DGK*δ* expression profiles. Hyperglycemia, a cardinal feature of type 2 diabetes, regulates DGK*δ* abundance and subcellular localization (Miele et al. 2007; Chibalin et al. 2008; Takeuchi et al. 2012), as well as DGK*δ* enzyme activity against palmitic acid-containing DAG species (Sakai et al. 2014). Interestingly saturated fatty acids increase and monounsaturated fatty acids attenuated DGK*δ* mRNA expression in C2C12 cells (Sakiyama et al. 2014). Consequently, the level of glycemia or different lipid species in type 2 diabetes or obesity may impact the abundance and activity of DGK*δ*. DGK*η* regulates a wide variety of physiological events including cell growth and proliferation. DGK*η* is linked to bipolar disorder (Moya et al. 2010), lung cancer (Nakano et al. 2014), and heart failure (Bilim et al. 2011), but its functional role in metabolic disorder is unclear. In *ob/ob* mice, DGK*η* was slightly reduced in soleus muscle and epididymal adipose tissue, while increased in subcutaneous adipose tissue. While DGK*δ* plays a role in metabolic disease, DGK*η* regulates other fundamental processes including proliferation and differentiation via Ras/B-Raf/C-Raf/MEK/ERK signaling (Yasuda et al. 2009). Dissecting the role of DGK*δ* and DGK*η* along diverse metabolic and growth-promoting pathways may further elucidate the diverse pathogenesis of chronic disorders including insulin resistance, heart failure, sarcopenia, and cancer.

DGK*ε* is the only member of the type III DGKs and is distinguished by a unique structure and substrate specificity toward arachidonate-containing DAG (Shulga et al. 2011). DGK*ε* knockout mice have a higher resistance to seizures induced by electroconvulsive shock (Rodriguez de Turco et al. 2001), while transgenic mice overexpressing DGK*ε* are protected from experimental cardiac hypertrophy (Niizeki et al. 2008). DGK*ε* is expressed in various immune cells, with increased levels observed in response to inflammatory stimuli (Yamamoto et al. 2014). In humans, DGK*ε* is highly expressed in brain and spleen, with lower, but comparable levels in liver, skeletal muscle and adipose tissue (Krupp et al. 2012). We observed DGK*ε* expression was marginally reduced in EDL muscle and appreciably reduced in epididymal adipose tissue from *ob/ob* mice. This was unexpected given that overexpression of DGK*ɛ* in muscle cells leads to defects in insulin signaling (Cazzolli et al. 2007).

The subfamily of type IV isoforms includes DGK*ζ* and DGK*ι*, which contain ankyrin repeats, a C-terminal nuclear localization signal and a PDZ-binding motif, as well as MARCKS homology region (Shulga et al. 2011). Analogous to DGK*α*, DGK*ζ* plays a critical role in immune cells (Zhong et al. 2003). However, DGK*ζ* is ubiquitously expressed in most tissues and most predominant in the brain (Krupp et al. 2012), suggesting a role in several organ systems. We found DGK*ζ* to be highly expressed in all insulin-sensitive tissues. DGK*ζ* expression was increased in epididymal adipose tissue and liver from *ob/ob* mice, while similar levels were noted in subcutaneous adipose tissue, soleus muscle and EDL muscle compared to C57BL/6J mice. The second member in the type IV DGK group, DGK*ι*, is predominately expressed in brain, but lowly expressed in insulin sensitivity tissues (Krupp et al. 2012). We found DGK*ι* mRNA expression was increased in soleus and EDL muscle, as well as subcutaneous and epididymal adipose tissue from *ob/ob* mice. The expression of DGK*ι* decreases in 3T3-L1 cells during adipocyte differentiation (Shulga et al. 2013). Interestingly, the National Heart, Lung, and Blood Institute Family Heart Study (FHS) genome-wide linkage scan identified DGK*ι* is a candidate gene for influencing BMI (Laramie et al. 2009), but secondary validation and functional studies are required to confirm this association.

DGK*θ*, the single member of the type V DGK, has three C1 domains, a Gly/Pro-rich domain and a pleckstrin homology domain (Shulga et al. 2011). Overexpression of DGK*θ* in mouse hepatocytes increases PA and decreases DAG content, while concomitantly impairing insulin signaling (Zhang et al. 2014). Consistent with other DGK isoforms, DGK*θ* is highly expressed in different regions of the brain in rats (Houssa et al. 1997). We observed that DGK*θ* mRNA was increased in EDL muscle, decreased in soleus muscle and unaltered in liver and adipose tissue depots in *ob/ob* versus lean mice. Nevertheless, DGK*θ* mRNA was lowly expressed compared to other isoforms, similar to RNA-Seq data from human tissues (Krupp et al. 2012). DGK*θ* has emerged as a relevant target for metabolic regulation given evidence that this isoforms acts as a key mediator of bile-acid-stimulated modulation PA-dependent mTOR and Akt signaling and glucose homoeostasis in HepG2 cells and primary human hepatocytes (Cai and Sewer 2013).

The identification of reference genes that are consistently expressed across several tissues is a frequent limitation, given the fact that even references genes often have a variable tissue-specific mRNA expression profile. To deal with this limitation, we profiled several reference genes expressed in the various metabolic tissues under study. Based on the results of this analysis, we were unable to identify a reference gene that was stable in all the tissues studied. Therefore, we present the C_q_ values and have normalized the data for the analysis comparing the expression of different DGK isoforms across the various tissues against the expression level in EDL muscle. Because we were unable to the normalize mRNA expression of the individual DGK isoforms between the various tissues to a common set of reference genes, we cannot exclude that this approach might have induced potential artifacts. When comparing mRNA expression profile of DGK isoforms within each tissue of C57BL/6J and *ob/ob* mice, the isoforms were normalized to the reference genes relevant for each tissue and then to a calibrator gene.

In summary, we provide evidence for tissue-specific expression profiles of DGK isoforms insulin-sensitive tissue from lean C57BL/6J mice. DGK*δ* is the most abundant isoform in soleus muscle, subcutaneous and epididymal adipose tissue. DGK*α* and DGK*ζ* are the predominant isoforms in EDL muscle. Finally, in liver, DGK*ζ* is the predominant isoform, with comparable levels of DGK*θ* and DGK*δ* noted. Overall, mRNA expression of DGK isoforms was generally lower in liver compared to skeletal muscle and adipose tissue. In conclusion, DGK expression was altered in an isoform- and tissue-specific manner in obese insulin-resistant *ob/ob* mice, suggesting DGKs play unique roles in each tissue and may play a role in metabolic disorders. Further studies are warranted to elucidate whether the altered DGK isoform expression profile in observed in obese insulin-resistant *ob/ob* mice has deleterious impact on tissue levels of PA and DAG as well as total DGK activity. Several DGK isoforms likely work in concert to modulate growth and metabolism in insulin sensitivity tissues.

## References

[b1] Bilim O, Shishido T, Toyama S, Suzuki S, Sasaki T, Kitahara T (2011). Differential regulation of diacylglycerol kinase isoform in human failing hearts. J. Cardiothorac. Surg.

[b2] Cai K, Sewer MB (2013). Diacylglycerol kinase theta couples farnesoid X receptor-dependent bile acid signalling to Akt activation and glucose homoeostasis in hepatocytes. Biochem. J.

[b3] Cazzolli R, Mitchell TW, Burchfield JG, Pedersen DJ, Turner N, Biden TJ (2007). Dilinoleoyl-phosphatidic acid mediates reduced IRS-1 tyrosine phosphorylation in rat skeletal muscle cells and mouse muscle. Diabetologia.

[b4] Chibalin AV, Leng Y, Vieira E, Krook A, Bjornholm M, Long YC (2008). Downregulation of diacylglycerol kinase delta contributes to hyperglycemia-induced insulin resistance. Cell.

[b5] Crotty T, Cai J, Sakane F, Taketomi A, Prescott SM, Topham MK (2006). Diacylglycerol kinase delta regulates protein kinase C and epidermal growth factor receptor signaling. Proc. Natl Acad. Sci. USA.

[b6] Dupuis J, Langenberg C, Prokopenko I, Saxena R, Soranzo N, Jackson AU (2010). New genetic loci implicated in fasting glucose homeostasis and their impact on type 2 diabetes risk. Nat. Genet.

[b7] Erion DM, Shulman GI (2010). Diacylglycerol-mediated insulin resistance. Nat. Med.

[b8] Houssa B, Schaap D, van der Wal J, Goto K, Kondo H, Yamakawa A (1997). Cloning of a novel human diacylglycerol kinase (DGK*θ*) containing three cysteine-rich domains, a proline-rich region, and a pleckstrin homology domain with an overlapping Ras-associating domain. J. Biol. Chem.

[b9] Hozumi Y, Matsui H, Sakane F, Watanabe M, Goto K (2013). Distinct expression and localization of diacylglycerol kinase isozymes in rat retina. J. Histochem. Cytochem.

[b10] Ishisaka M, Hara H (2014). The roles of diacylglycerol kinases in the central nervous system: review of genetic studies in mice. J. Pharmacol. Sci.

[b11] Ishisaka M, Kakefuda K, Oyagi A, Ono Y, Tsuruma K, Shimazawa M (2012). Diacylglycerol kinase beta knockout mice exhibit attention-deficit behavior and an abnormal response on methylphenidate-induced hyperactivity. PLoS One.

[b12] Katagiri Y, Ito T, Saino-Saito S, Hozumi Y, Suwabe A, Otake K (2005). Expression and localization of diacylglycerol kinase isozymes and enzymatic features in rat lung. Am. J. Physiol. Lung Cell. Mol. Physiol.

[b13] Krishna S, Zhong XP (2013). Regulation of lipid signaling by diacylglycerol kinases during T cell development and function. Front Immunol.

[b14] Krupp M, Marquardt JU, Sahin U, Galle PR, Castle J, Teufel A (2012). RNA-Seq Atlas—a reference database for gene expression profiling in normal tissue by next-generation sequencing. Bioinformatics.

[b15] Kurohane Kaneko Y, Kobayashi Y, Motoki K, Nakata K, Miyagawa S, Yamamoto M (2013). Depression of type I diacylglycerol kinases in pancreatic beta-cells from male mice results in impaired insulin secretion. Endocrinology.

[b16] Laramie JM, Wilk JB, Williamson SL, Nagle MW, Latourelle JC, Tobin JE (2009). Multiple genes influence BMI on chromosome 7q31-34: the NHLBI Family Heart Study. Obesity (Silver Spring).

[b17] Livak KJ, Schmittgen TD (2001). Analysis of relative gene expression data using real-time quantitative PCR and the 2-ΔΔCT method. Methods.

[b18] Lowe CE, Zhang Q, Dennis RJ, Aubry EM, O'Rahilly S, Wakelam MJ (2013). Knockdown of diacylglycerol kinase delta inhibits adipocyte differentiation and alters lipid synthesis. Obesity (Silver Spring).

[b19] Miele C, Paturzo F, Teperino R, Sakane F, Fiory F, Oriente F (2007). Glucose regulates diacylglycerol intracellular levels and protein kinase C activity by modulating diacylglycerol kinase subcellular localization. J. Biol. Chem.

[b20] Moya PR, Murphy DL, McMahon FJ, Wendland JR (2010). Increased gene expression of diacylglycerol kinase eta in bipolar disorder. Int. J. Neuropsychopharmacol.

[b21] Nakano T, Hozumi Y, Iwazaki K, Okumoto K, Iseki K, Saito T (2012). Altered expression of diacylglycerol kinase isozymes in regenerating liver. J. Histochem. Cytochem.

[b22] Nakano T, Iravani A, Kim M, Hozumi Y, Lohse M, Reichert E (2014). Diacylglycerol kinase eta modulates oncogenic properties of lung cancer cells. Clin. Transl. Oncol.

[b23] Niizeki T, Takeishi Y, Kitahara T, Arimoto T, Ishino M, Bilim O (2008). Diacylglycerol kinase-epsilon restores cardiac dysfunction under chronic pressure overload: a new specific regulator of Galpha(q) signaling cascade. Am. J. Physiol. Heart Circ. Physiol.

[b24] Olenchock BA, Guo R, Carpenter JH, Jordan M, Topham MK, Koretzky GA (2006). Disruption of diacylglycerol metabolism impairs the induction of T cell anergy. Nat. Immunol.

[b25] Regier DS, Higbee J, Lund KM, Sakane F, Prescott SM, Topham MK (2005). Diacylglycerol kinase iota regulates Ras guanyl-releasing protein 3 and inhibits Rap1 signaling. Proc. Natl Acad. Sci. USA.

[b26] Rodriguez de Turco EB, Tang W, Topham MK, Sakane F, Marcheselli VL, Chen C (2001). Diacylglycerol kinase epsilon regulates seizure susceptibility and long-term potentiation through arachidonoyl- inositol lipid signaling. Proc. Natl Acad. Sci. USA.

[b27] Sakai H, Sakane F (2012). Recent progress on type II diacylglycerol kinases: the physiological functions of diacylglycerol kinase delta, eta and kappa and their involvement in disease. J. Biochem.

[b28] Sakai H, Kado S, Taketomi A, Sakane F (2014). Diacylglycerol kinase delta phosphorylates phosphatidylcholine-specific phospholipase C-dependent, palmitic acid-containing diacylglycerol species in response to high glucose levels. J. Biol. Chem.

[b29] Sakiyama S, Usuki T, Sakai H, Sakane F (2014). Regulation of diacylglycerol kinase delta2 expression in C2C12 skeletal muscle cells by free fatty acids. Lipids.

[b30] Samuel VT, Shulman GI (2012). Mechanisms for insulin resistance: common threads and missing links. Cell.

[b31] Shionoya T, Usuki T, Komenoi S, Isozaki T, Sakai H, Sakane F (2015). Distinct expression and localization of the type II diacylglycerol kinase isozymes inverted question mark, inverted question mark and inverted question mark in the mouse reproductive organs. BMC Dev. Biol.

[b32] Shirai Y, Saito N (2014). Diacylglycerol kinase as a possible therapeutic target for neuronal diseases. J. Biomed. Sci.

[b33] Shirai Y, Kouzuki T, Kakefuda K, Moriguchi S, Oyagi A, Horie K (2010). Essential role of neuron-enriched diacylglycerol kinase (DGK), DGKbeta in neurite spine formation, contributing to cognitive function. PLoS One.

[b34] Shulga YV, Topham MK, Epand RM (2011). Regulation and functions of diacylglycerol kinases. Chem. Rev.

[b35] Shulga YV, Loukov D, Ivanova PT, Milne SB, Myers DS, Hatch GM (2013). Diacylglycerol kinase delta promotes lipogenesis. Biochemistry.

[b36] Takeuchi M, Sakiyama S, Usuki T, Sakai H, Sakane F (2012). Diacylglycerol kinase delta1 transiently translocates to the plasma membrane in response to high glucose. Biochim. Biophys. Acta.

[b37] Topham MK (2006). Signaling roles of diacylglycerol kinases. J. Cell. Biochem.

[b38] Toya M, Hozumi Y, Ito T, Takeda M, Sakane F, Kanoh H (2005). Gene expression, cellular localization, and enzymatic activity of diacylglycerol kinase isozymes in rat ovary and placenta. Cell Tissue Res.

[b39] Yamamoto M, Tanaka T, Hozumi Y, Saino-Saito S, Nakano T, Tajima K (2014). Expression of mRNAs for the diacylglycerol kinase family in immune cells during an inflammatory reaction. Biomed. Res.

[b40] Yasuda S, Kai M, Imai S, Takeishi K, Taketomi A, Toyota M (2009). Diacylglycerol kinase eta augments C-Raf activity and B-Raf/C-Raf heterodimerization. J. Biol. Chem.

[b41] Zachariah Tom R, Garcia-Roves PM, Sjögren RJO, Jiang LQ, Holmström MH, Deshmukh AS (2014). Effects of AMPK Activation on Insulin Sensitivity and Metabolism in Leptin-Deficient ob/ob Mice. Diabetes.

[b42] Zhang C, Klett EL, Coleman RA (2013). Lipid signals and insulin resistance. Clin. Lipidol.

[b43] Zhang C, Hwarng G, Cooper DE, Grevengoed TJ, Eaton JM, Natarajan V (2014). Inhibited insulin signaling in mouse hepatocytes is associated with increased phosphatidic acid but not diacylglycerol. J. Biol. Chem.

[b44] Zhong XP, Hainey EA, Olenchock BA, Jordan MS, Maltzman JS, Nichols KE (2003). Enhanced T cell responses due to diacylglycerol kinase zeta deficiency. Nat. Immunol.

